# Supplementary material for: Remote Activation of a Latent Epitope in an Autoantigen Decoded With Simulated B-Factors
Source: Front Immunol. 2019 Oct 25;10:2467. doi: 10.3389/fimmu.2019.02467 (PMC6823208; doi:10.3389/fimmu.2019.02467)

Remote Activation of a Latent Epitope in an Autoantigen Decoded with Simulated B-Factors

Yuan-Ping Pang[1]*, Marta Casal Moura[2], Gwen E. Thompson[2], Darlene R. Nelson[2], Amber M. Hummel[2], Dieter E. Jenne[3,4], Daniel Emerling[5], Wayne Volkmuth[5], William H. Robinson[6], and Ulrich Specks[2]*

[1] Computer-Aided Molecular Design Laboratory, Mayo Clinic, Rochester, MN, USA, [2] Thoracic Disease Research Unit, Mayo Clinic, Rochester, MN, USA, [3] Institute of Lung Biology and Disease, Helmholtz-Center, Munich, Germany, [4] Max Planck Institute of Neurobiology, Planegg-Martinsried, Germany, [5] Atreca, Inc., Redwood City, CA, USA, [6] Stanford University, Palo Alto, CA, USA

***Correspondence:**

Yuan-Ping Pang

pang@mayo.edu

Ulrich Specks

specks.ulrich@mayo.edu

Table S1. Alpha carbon root mean square deviations (Å) among different PR3 variants

	Xray-PR3-Ile[103]	Comp-PR3-Ile[103]	Comp-PR3-Val[103]	Com-iHm5-Val[103]
Com-PR3-Ile[103]	1.67	0	0.62	1.63
Com-PR3-Val[103]	1.90	0.62	0	1.56
Com-iPR3-Val[103]	1.75	0.59	0.45	1.41
Com-iHm5-Val[103]	2.34	1.63	1.56	0

Table S2. Alpha carbon B-factors of three PR3 variants

residue ID[a]	PR3-Val[119]		iPR3-Val[119]		iHm5-Val[119]	
	mean (n = 20)	SEM[b]	mean (n = 20)	SEM[b]	mean (n = 20)	SEM[b]
28	3.56	0.31	5.60	0.44	4.79	0.31
29	3.93	0.31	5.40	0.31	5.39	0.42
30	7.28	0.80	8.64	0.58	7.62	0.49
31	6.34	0.39	8.77	1.01	5.92	0.38
32	6.20	0.41	6.97	0.45	5.84	0.34
33	5.39	0.24	6.79	0.64	4.65	0.28
34	5.72	0.29	6.57	0.53	4.91	0.25
35	8.46	0.85	9.14	1.03	7.17	0.56
36	9.37	0.83	8.96	0.46	7.22	0.49
37	7.06	0.67	6.86	0.32	6.35	0.33
38	6.24	0.43	5.79	0.63	8.11	0.47
39	6.44	0.44	6.33	0.44	5.83	0.41
40	7.26	0.84	6.17	0.73	4.83	0.31
41	3.94	0.18	3.46	0.15	3.17	0.15
42	4.17	0.27	4.21	0.37	3.73	0.13
43	4.52	0.23	4.30	0.22	3.29	0.12
44	3.72	0.17	3.62	0.13	3.16	0.18
45	3.39	0.13	3.40	0.14	3.59	0.19
46	4.41	0.22	4.16	0.16	5.08	0.29
47	8.26	0.57	6.51	0.27	6.90	0.38
48	10.26	0.67	7.96	0.40	9.97	0.49
49	23.33	1.73	18.49	1.40	17.91	1.18
50	17.78	1.86	14.51	0.97	14.97	1.03
51	10.48	1.01	14.52	1.23	17.56	2.29
52	10.62	0.67	13.58	1.51	16.14	1.72
53	7.79	0.46	7.08	0.46	10.28	0.87
54	5.53	0.32	4.99	0.19	6.03	0.27
55	5.30	0.31	5.39	0.30	5.25	0.34
56	4.61	0.26	4.59	0.30	5.24	0.35
57	4.93	0.37	4.10	0.16	4.67	0.30
58	5.33	0.22	4.54	0.30	4.84	0.22
59	3.50	0.14	3.33	0.11	3.04	0.14
60	2.61	0.10	2.45	0.07	2.34	0.08
61	3.52	0.15	3.39	0.09	3.60	0.15
62	4.36	0.18	4.29	0.15	4.49	0.19
63	5.70	0.31	5.46	0.15	5.26	0.26
64	5.85	0.43	5.12	0.22	3.62	0.15
65	3.25	0.10	3.09	0.08	2.86	0.09
66	2.83	0.08	2.68	0.06	2.54	0.07
67	2.94	0.11	2.86	0.07	3.02	0.13
68	3.36	0.13	3.31	0.13	2.94	0.09
69	3.92	0.20	3.99	0.22	3.62	0.14
70	3.49	0.13	3.76	0.20	3.31	0.12
71	4.29	0.17	4.57	0.25	4.12	0.17
72	6.95	0.40	7.28	0.60	5.89	0.25

73	7.06	0.46	8.78	0.88	5.71	0.32
74	11.84	1.54	13.07	1.37	9.68	0.71
75	19.26	3.66	13.92	1.17	12.93	1.61
76	16.13	2.05	10.37	0.83	9.06	0.67
77	13.62	0.92	10.58	0.68	9.46	0.52
78	12.19	1.25	11.60	1.10	11.22	0.81
79	12.90	1.20	11.60	1.30	11.54	0.90
80	9.70	0.49	9.29	0.86	8.66	0.64
81	7.88	0.44	6.25	0.27	5.40	0.29
82	5.56	0.31	5.62	0.29	4.25	0.22
83	3.76	0.12	3.61	0.11	3.18	0.13
84	3.26	0.15	3.07	0.10	2.95	0.15
85	3.69	0.26	3.44	0.16	3.09	0.16
86	5.63	0.58	4.26	0.24	4.90	0.25
87	5.91	0.76	5.67	0.98	5.28	0.40
88	4.29	0.26	4.06	0.17	4.78	0.20
89	3.86	0.15	4.03	0.15	4.57	0.23
90	4.58	0.22	4.95	0.28	5.02	0.27
91	7.12	0.41	7.07	0.47	8.71	0.70
92	8.36	0.47	9.57	0.72	12.59	0.85
93	7.85	0.55	8.27	0.50	13.56	1.13
94	9.76	0.68	8.41	0.46	7.92	0.54
95	14.44	1.18	11.30	0.57	12.23	1.80
96	10.63	1.08	7.91	0.33	7.02	0.63
97	5.87	0.48	4.82	0.17	7.95	1.01
98	5.29	0.32	5.04	0.30	4.68	0.31
99	5.57	0.27	5.78	0.32	4.39	0.25
100	4.80	0.20	4.76	0.20	3.96	0.14
101	6.34	0.30	4.95	0.19	5.82	0.29
102	5.86	0.26	5.37	0.18	5.91	0.24
103	6.57	0.24	7.39	0.49	7.53	0.35
104	6.49	0.42	6.67	0.39	7.42	0.34
105	5.91	0.33	5.47	0.23	6.26	0.33
106	5.23	0.22	5.35	0.25	5.16	0.26
107	6.98	0.44	7.73	0.59	7.05	0.67
108	8.72	0.59	7.81	0.71	11.90	1.93
109	11.35	0.92	11.15	0.94	13.49	1.65
110	8.06	0.56	7.27	0.48	8.06	0.48
111	8.89	0.68	8.23	0.55	8.43	0.56
112	13.91	1.28	12.71	0.76	11.24	0.80
113	16.56	1.98	17.66	1.51	13.15	1.30
114	12.60	1.61	12.03	0.94	9.89	0.91
115	8.34	0.71	6.99	0.48	6.01	0.54
116	6.25	0.41	5.03	0.22	5.02	0.30
117	4.50	0.18	4.52	0.20	3.82	0.23
118	3.23	0.11	3.47	0.18	2.89	0.09
119	3.66	0.14	3.93	0.22	3.67	0.14
120	3.36	0.10	3.67	0.16	3.73	0.13
121	3.22	0.09	3.41	0.10	3.09	0.09
122	3.47	0.13	3.70	0.11	3.46	0.13
123	4.15	0.14	4.20	0.12	3.59	0.13
124	5.12	0.15	4.80	0.19	3.90	0.18
125	8.16	0.50	6.83	0.39	6.39	0.22
126	10.38	0.70	10.26	0.62	7.87	0.49
127	9.01	0.69	9.11	0.45	7.21	0.35
128	8.03	0.47	7.94	0.52	5.29	0.33
129	10.87	1.10	9.77	0.91	7.57	1.58
130	9.92	0.95	8.53	0.37	9.28	0.75
131	22.48	2.73	17.61	1.38	16.09	2.31

132	23.75	2.39	20.77	2.37	14.38	2.82
133	17.51	2.06	10.80	0.99	10.93	1.03
134	8.05	0.71	6.19	0.40	5.28	0.36
135	6.99	0.60	6.16	0.27	5.05	0.44
136	4.47	0.23	3.70	0.13	4.28	0.45
137	3.97	0.14	3.43	0.13	4.43	0.46
138	5.55	0.28	4.61	0.19	6.01	0.67
139	6.11	0.25	5.63	0.22	6.82	0.50
140	5.20	0.10	5.05	0.13	8.03	0.39
141	7.71	0.44	5.99	0.21	15.44	1.57
142	6.64	0.49	5.53	0.30	14.24	2.08
143	6.23	0.34	5.21	0.26	11.31	1.93
144	7.86	0.56	5.00	0.26	12.60	1.62
145	8.66	0.62	7.42	0.36	15.05	1.19
146	8.00	0.46	6.31	0.38	10.03	0.53
147	9.72	0.41	9.90	0.52	14.43	1.09
148	7.77	0.27	9.51	0.56	12.73	1.15
149	7.67	0.40	9.45	0.46	13.90	1.87
150	5.96	0.28	6.73	0.38	7.17	0.53
151	5.16	0.23	5.62	0.36	6.43	0.31
152	3.70	0.10	3.45	0.09	4.13	0.13
153	3.31	0.14	3.49	0.10	4.02	0.13
154	3.49	0.09	3.90	0.11	4.02	0.10
155	3.88	0.10	4.21	0.20	4.89	0.17
156	5.73	0.52	6.15	0.47	6.57	0.47
157	3.84	0.31	4.39	0.31	3.76	0.29
158	4.92	0.29	6.94	0.57	5.34	0.65
159	4.89	0.25	5.95	0.44	5.21	0.37
160	6.75	0.38	10.46	1.45	6.11	0.34
161	9.83	0.70	14.55	2.18	11.41	1.02
162	14.05	1.29	20.99	2.59	12.20	1.01
163	18.35	1.74	24.75	2.29	15.93	1.11
164	13.99	1.82	14.99	1.15	13.31	0.95
165	13.27	1.31	12.36	0.68	13.28	1.33
166	9.76	1.20	8.31	0.49	9.26	0.87
167	6.25	0.79	7.30	0.47	7.33	0.43
168	5.12	0.35	5.42	0.32	5.96	0.32
169	4.23	0.19	3.93	0.12	3.78	0.15
170	3.32	0.14	3.34	0.10	4.06	0.27
171	3.03	0.13	3.79	0.50	3.66	0.24
172	3.14	0.15	3.18	0.20	3.31	0.12
173	3.82	0.19	4.54	0.18	4.24	0.24
174	4.96	0.29	4.87	0.21	4.80	0.23
175	4.34	0.28	3.88	0.26	3.83	0.18
176	3.81	0.14	4.26	0.25	4.19	0.18
177	3.99	0.18	4.24	0.26	3.96	0.13
178	5.56	0.24	5.50	0.29	5.26	0.25
179	7.24	0.66	6.72	0.41	7.75	0.61
180	10.82	1.03	11.74	0.69	14.32	1.44
181	12.04	0.88	14.22	1.21	12.05	0.87
182	7.22	0.62	8.24	0.49	6.87	0.44
183	6.00	0.29	7.27	0.50	6.76	0.56
184	6.81	0.42	8.31	0.56	7.53	0.53
185	3.81	0.18	4.57	0.36	3.94	0.23
186	3.60	0.17	3.63	0.13	3.16	0.19
187	3.42	0.10	3.26	0.13	3.33	0.13
188	3.96	0.26	4.26	0.29	3.93	0.16
189	3.81	0.21	4.40	0.38	4.17	0.19
190	4.72	0.33	5.32	0.79	4.78	0.18

191	5.48	0.31	6.89	0.84	5.86	0.38
192	11.37	0.89	13.69	2.05	11.01	1.22
193	13.67	1.31	15.15	2.30	10.60	0.93
194	10.60	0.61	10.83	1.01	12.10	1.10
195	8.73	0.49	10.45	0.87	12.66	1.31
196	5.13	0.27	6.91	0.63	8.07	0.66
197	4.28	0.14	6.04	0.56	5.16	0.26
198	3.83	0.18	4.65	0.41	3.91	0.13
199	4.62	0.25	5.99	0.37	4.58	0.21
200	6.13	0.52	11.85	1.40	7.84	0.47
201	7.88	0.56	15.72	1.54	9.81	1.02
202	3.86	0.28	6.30	0.57	4.41	0.36
203	4.11	0.22	7.41	1.22	5.45	0.36
204	5.56	0.52	5.07	0.28	4.18	0.20
205	5.21	0.30	5.17	0.36	4.71	0.20
206	3.35	0.11	3.33	0.14	3.27	0.09
207	2.97	0.10	2.78	0.09	2.69	0.07
208	3.26	0.08	2.91	0.09	3.55	0.16
209	4.98	0.18	4.47	0.14	5.22	0.28
210	12.93	0.64	11.47	0.78	12.09	0.96
211	12.26	0.63	8.85	0.41	11.09	0.57
212	4.44	0.14	3.81	0.12	4.78	0.22
213	3.34	0.13	3.02	0.06	3.54	0.21
214	2.85	0.09	2.70	0.06	3.11	0.17
215	4.83	0.23	4.78	0.25	4.89	0.25
216	3.15	0.22	3.40	0.19	3.14	0.11
217	3.69	0.40	3.58	0.19	3.13	0.10
218	4.01	0.27	3.77	0.24	3.16	0.11
219	4.77	0.28	4.42	0.20	3.85	0.16
220	5.24	0.42	5.76	0.45	5.35	0.24
221	8.22	0.59	7.06	0.47	6.56	0.48
222	9.35	0.81	12.41	1.09	6.07	0.38
223	10.59	0.80	14.58	1.43	7.32	0.40
224	6.13	0.33	7.99	0.58	8.49	0.46
225	5.73	0.25	7.11	0.50	16.39	0.92
226	7.81	0.43	6.82	0.67	22.29	3.15
227	7.29	0.36	8.37	0.93	11.36	0.83
228	9.81	0.53	10.33	1.31	14.41	2.07
229	7.50	0.40	7.78	0.75	11.17	1.10
230	4.84	0.24	5.40	0.67	6.98	0.51
231	3.18	0.17	4.01	0.67	3.93	0.19
232	3.35	0.14	4.33	0.64	3.24	0.11
233	2.88	0.12	2.96	0.10	2.65	0.08
234	2.63	0.10	3.00	0.14	2.58	0.07
235	2.90	0.10	2.82	0.09	3.10	0.10
236	3.33	0.10	3.27	0.12	4.87	0.30
237	4.13	0.15	3.93	0.16	7.64	0.65
238	4.38	0.16	4.51	0.18	6.99	0.49
239	4.74	0.22	5.06	0.21	6.91	0.53
240	4.98	0.30	4.83	0.17	8.61	0.74
241	8.03	0.65	7.22	0.37	10.68	0.73
242	7.29	0.60	7.39	0.31	12.37	1.07
243	7.81	0.68	8.50	0.64	25.91	2.54

[a]The residue numbering here is identical to that of the human PR3 sequence (NCBI P24158.3).

[b]SEM: Standard error of the mean.

Figure S1. Western blots. B.1. Comparable binding of the murine anti-c-myc moAb (1.0 μg/mL) to the C-terminal cmyc-tag of the two antigens. B.2. Binding of moANCA518 (0.5 μg/mL) to iHm5-Val^{103} only.

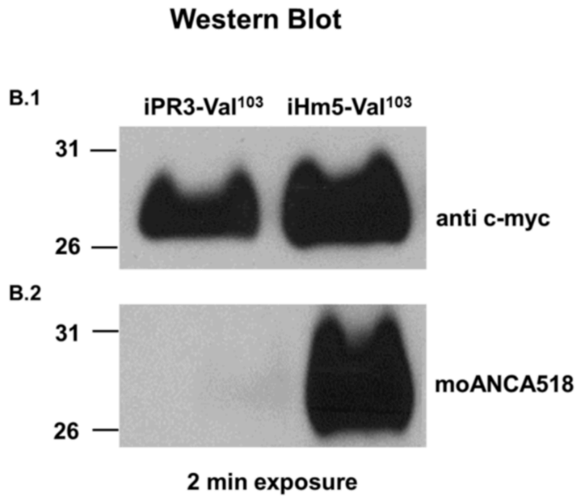

Supplement: Supplementary file 1 [file Data_Sheet_1.PDF]
